# A novel paclitaxel eluting bioresorbable vascular stent with a super flexible stent structure and round cross section struts fabricated using 3D printing technology with a rotating platform

**DOI:** 10.1093/rb/rbaf073

**Published:** 2025-07-09

**Authors:** Wei Liu, Qingqing Li, Ge Song, Zhiqi Lin, Xiaofei Gong, Hanqing Feng, Hugh Q Zhao, Yujie Zhou, Yunbing Wang, Zhongyong Fan, Qing Liu

**Affiliations:** Cardiovascular Department, Jishuitan Hospital, Beijing, 100035, P. R. China; Beijing Advanced Medical Technologies, Ltd Inc, Beijing 102600, P. R. China; Beijing Advanced Medical Technologies, Ltd Inc, Beijing 102600, P. R. China; Beijing Advanced Medical Technologies, Ltd Inc, Beijing 102600, P. R. China; Beijing Advanced Medical Technologies, Ltd Inc, Beijing 102600, P. R. China; Beijing Advanced Medical Technologies, Ltd Inc, Beijing 102600, P. R. China; Beijing Advanced Medical Technologies, Ltd Inc, Beijing 102600, P. R. China; College of Biomedical Engineering, Sichuan University, Chengdu, Sichuan, 610065, P. R. China; Beijing Anzhen Hospital, Capital Medical University, Beijing, 100029, P.R. China; College of Biomedical Engineering, Sichuan University, Chengdu, Sichuan, 610065, P. R. China; Department of Materials Science, Fudan University, Shanghai 200433, P. R. China; Beijing Advanced Medical Technologies, Ltd Inc, Beijing 102600, P. R. China

**Keywords:** coronary stent, bioresorbable stent, paclitaxel drug release, poly(L-lactide-co-ɛ-caprolactone) copolymer, 3D printing

## Abstract

Bioresorbable stents (BRS) have emerged as a groundbreaking development in the field of percutaneous coronary intervention (PCI) as they address the long-standing concerns of metallic stents. Nevertheless, the observed higher thrombosis rates in the first generation BRS, i.e. ABSORB^®^, might be attributed to their thicker struts, slower degradation rate and structural dismantling of partially endothelialized stents. In this study, measures have been taken to overcome these limitations include reducing strut thickness, modifying the structural design to maintain radial strength with thinner round cross section struts and using a new material poly(L-lactide-co-ɛ-caprolactone) (PLCL 95/5) that is tougher and degrade faster than poly(L-lactic acid) (PLLA).Given the excellent biocompatibility of PLCL materials, the US FDA has approved their use in clinical applications. PLCL stents can be used to treat diseases such as tracheal stenosis and tracheoesophageal fistula, and can also be applied in the construction of other tissue engineering stents, such as nerve conduitsand fat filling stents. The newly designed coronary stents were fabricated using a 3D printing technology with a rotating platform, coated with a paclitaxel coating and comprehensive in vitro research was conducted. It was the first to undergo tests in animals. Results showed the novel paclitaxel eluting PLCL stents had super-flexible structure, thinner round cross-sectional struts, a faster degradation profile and satisfactory hemocompatibility. With a paclitaxel dose of 0.57 μg/mm^2^, the drug eluting stents showed very low degree of stenosis within 6 months of implantation in a porcine model. Overall, the results showed that the novel 3D printed PLCL drug eluting stent is a very promising candidate for next generation bioresorbable coronary stent.

## Graphical Abstract

**Figure:**
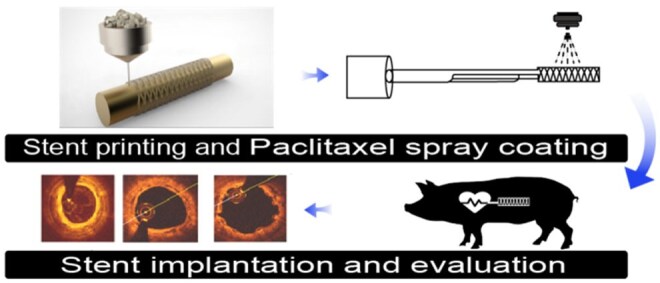

## Introduction

Bioresorbable stents (BRS) have emerged as a groundbreaking development in the field of percutaneous coronary intervention (PCI) as they address the long-standing concerns of metallic stents in the coronary artery, such as the caging of the vessel permanently with the permanent metallic implant that impair endothelial function and decrease positive lumen remodeling. Unlike traditional metallic stents, BRS have the potential to restore normal vasomotion and completely dissolve over time, leaving no permanent implant behind [1–3].

However, the initial clinical results of the first FDA-approved polymeric BRS, ABSORB^®^ BVS (Abbott Vascular Inc.), proved to be inferior to metallic drug-eluting stents Xience V, leading to its withdrawal from the market in 2017 [4, 5]. ABSORB^®^ BVS appeared to exhibit higher rates of acute (1–2 days) and very-late stent thrombosis (3–5 years) after implantation [6–8]. This increased risk of thrombosis was attributed to the insufficient neointimal-stent integration which allowed fractured struts to protrude from the vessel wall into the lumen, triggering thrombosis. Several stent failure mechanisms were proposed. Among these, thicker strut and structural dismantling at the non-endothelialized part of the stent were identified as the device related factors. Thicker struts might not only negatively affect the endothelization process as mentioned above but also cause disturbance to the blood flow, and thus, increase the risk of in-stent thrombosis. Toong *et al*. [9] has shown that thicker struts can cause shear/laminar blood flow disruptions, reduce endothelialization and increase thrombogenicity. Therefore, newer generation BRS should focus on reducing strut thickness, improving the mechanical properties to minimize stent recoil, exploration of new materials to prevent stent discontinuity with faster resorption rate [10, 11].

Reducing the strut thickness of the stent without changing the stent material and/or stent structure is very challenging because the mechanical strength of a polymer is inferior to that of a metallic material. If use the same stent design and material, simply reducing the strut thickness will not work because it will reduce the radial strength, which is essential to supporting the vessels. Therefore, changing the stent structure design is both a logical and practical way when strut thickness needs to be reduced. Additionally, changing the structure design is an effective way to improve the flexibility of the stent. Flexibility is one of the important characteristics of stent design. Good flexibility will allow stent pass through the torturous vessel successfully under the guidance of the catheter system, and prevent stent malposition after expansion, reducing the mechanical damage to blood vessel walls [12].

The stent geometrical design is one of the very important factors that affect the stent performance [13]. Therefore, structural optimization of stent design is necessary to find the optimal stent geometry design. One of the aspects of the stent structure is the shape of the strut cross section. A stent fabricated using a laser cutting technology typical has rectangular cross-section struts. The stent strut will affect the blood flow in terms of shear stress that in turn will affect the aggregation of red blood cells, platelet deformation and activation or in other words, the in-stent thrombosis [6]. Compared with a typical rectangular cross-sectional strut, a round cross-section struts may offer a solution in reducing the shear stress of the blood flow. It has been proposed that a round cross-sectional strut, after being embedding into vessel walls when stents are deployed using a balloon catheter, were believed to cause streamlined blood flow and thus lower shear stress, promotes rapid and complete re-endothelialization of the stent [14]. In our previous studies, we have found that 3D printed bioresorbable PLLA stents with round cross-section struts indeed showed fast endothelization in animals [15]. Furthermore, the 3D printed BRS showed a long-term positive remodeling effect in the porcine model. It also showed similar safety and efficacy to that of a marketed sirolimus eluting metallic stents [16].

In searching for better stent materials, several types of materials have been explored. Magnesium-based BRSs could provide better mechanical strength than polymeric stents. However, they have a faster degradation rate than polymeric devices in environments with chloride (blood), leading to a higher probability of adverse biological events after implantation [17]. Thus, future research should focus on the ways to improve the mechanical properties of bioresorbable polymeric stents due to their materials with higher cellular compatibility and suitable or adjustable degradation time compared with metallic materials [18]. New materials that are tougher and degrade faster than poly(L-lactic acid) (PLLA) were suggested [11]. Some new materials including citrate-based biomaterials (CBBs) [19], tyrosine derived polycarbonate (PC) (from REVA Medical [3]), poly(trimethylene carbonate-co-D, L-lactic acid)copolymer (P(TMC-co-DLLA)) [20], poly(p-dioxanone) (PPDO) and polycaprolactone (PCL) composites [21]. A copolymer of L-lactide and ɛ-caprolactone (PLCL) was also explored both in our lab and another lab and showed encouraging bench-top testing results [22, 23]. In terms of biocompatibility and antithrombotic properties, PLCL materials are more competitive than PLLA [24].

One of the emerging technologies in fabricating bioresorbable stents is 3D printing. This 3D printing technology is well known for its capability to produce personalized object and/or complex structures that traditional manufacturing technology could not [15, 19, 22, 25–33]. Therefore, 3D printing is a very powerful tool to produce bioresorbable stents with novel structure designs which may offer better performance in terms of radial strength and flexibility. A 3D printed paclitaxel eluting peripheral bioresorbable stent developed by our company is currently in clinical trial in China [34].

Regarding the drug selection for intravascular stents, there are usually paclitaxel, rapamycin and its derivatives, antiplatelet drugs and anti-inflammatory drugs. Taxol, naturally extracted from the bark and leaves of *Taxus chinensis*, was initially developed as an anticancer drug to inhibit tumor growth by suppressing microtubule polymerization and blocking cell mitosis. However, its unique antiproliferative properties have also been found to be applicable in the cardiovascular field, especially in preventing vascular restenosis. The mechanism of action of paclitaxel in cardiovascular stents is usually divided into two aspects. One is to inhibit the proliferation of vascular smooth muscle cells: Paclitaxel inhibits microtubule depolymerization, disrupts microtubule homeostasis and prevents cell division (M/G2 phase arrest) by binding to the alpha and beta subunits of microtubule proteins, thereby suppressing excessive proliferation and migration of smooth muscle cells after vascular injury [35, 36]. Its lipophilicity allows it to quickly penetrate deep into the blood vessel wall, persistently inhibiting the formation of neointima, while potentially promoting endothelial cell clearance and accelerating surface healing. This mechanism can effectively reduce restenosis caused by intimal hyperplasia after stent implantation. The second is local drug release: In cardiovascular devices, paclitaxel is released through drug-eluting stents (DES) or drug-coated balloons (DCB). The polymer coating of the stent can slowly release the drug to the vascular wall, while the balloon evenly applies the drug to the surface of the vascular endothelium through mechanical action during dilation.

Early paclitaxel DCB has been questioned due to controversy over long-term mortality, but recent studies (such as SCAAR registry studies) have shown its safety is controllable, especially in ISR treatment where it performs exceptionally well [37]. At present, paclitaxel is widely used in drug-eluting balloons (DCBs) and stents for coronary artery intervention (PCI), such as Boston Scientific Agent DCB, which is the world’s first FDA approved coronary DCB. It uses TransPax coating technology to target the release of paclitaxel and is suitable for in stent restenosis (ISR) and small vessel disease in coronary arteries. Approved in China in May 2024, and obtained CE, FDA and NMPA certifications. Yinyi Biotech Bingo DCB is the first approved DCB in China (2017), which uses polymer free micro blind hole drug delivery technology and is suitable for primary bifurcation lesions, covering over a thousand hospitals. Medtronic Prevail DCB adopts FreePac coating (paclitaxel + urea) combined with PowerTrac hydrophilic coating technology. Obtained FDA IDE approval in October 2024 and is currently conducting critical clinical trials. Yinyi Biotech’s polymer free micro blind hole drug loaded stent is a metal surface micro blind hole (pore size 12 μm) loaded with paclitaxel, without the need for a polymer carrier, reducing the risk of delayed endothelial healing. It is applicable to patients with diabetes, coronary heart disease and rapamycin allergy.

In this paper, 3D printed coronary stents with paclitaxel elution coating were fabricated from the copolymer of L-lactide and ɛ-caprolactone (PLCL 95/5) and were studied both *in vitro* and *in vivo*. Specifically, a proprietary four-axis 3D printing system was used for the fabrication of the totally bioresorbable PLCL polymer stents [15, 22, 29–32]. An abluminal paclitaxel-eluting coating was applied. Additionally, a new flexible stent structure design was utilized aimed at reducing strut thickness while still maintaining sufficient radial strength of the stents [32]. Since the stent struts were extruded from a 3D printing nozzle, the struts have round cross-sections and could be embedded into the vessel walls under high balloon expansion pressure, resulting in reduced blood flow disturbance and faster endothelialization [22].

## Materials and methods

**Figure 1.**
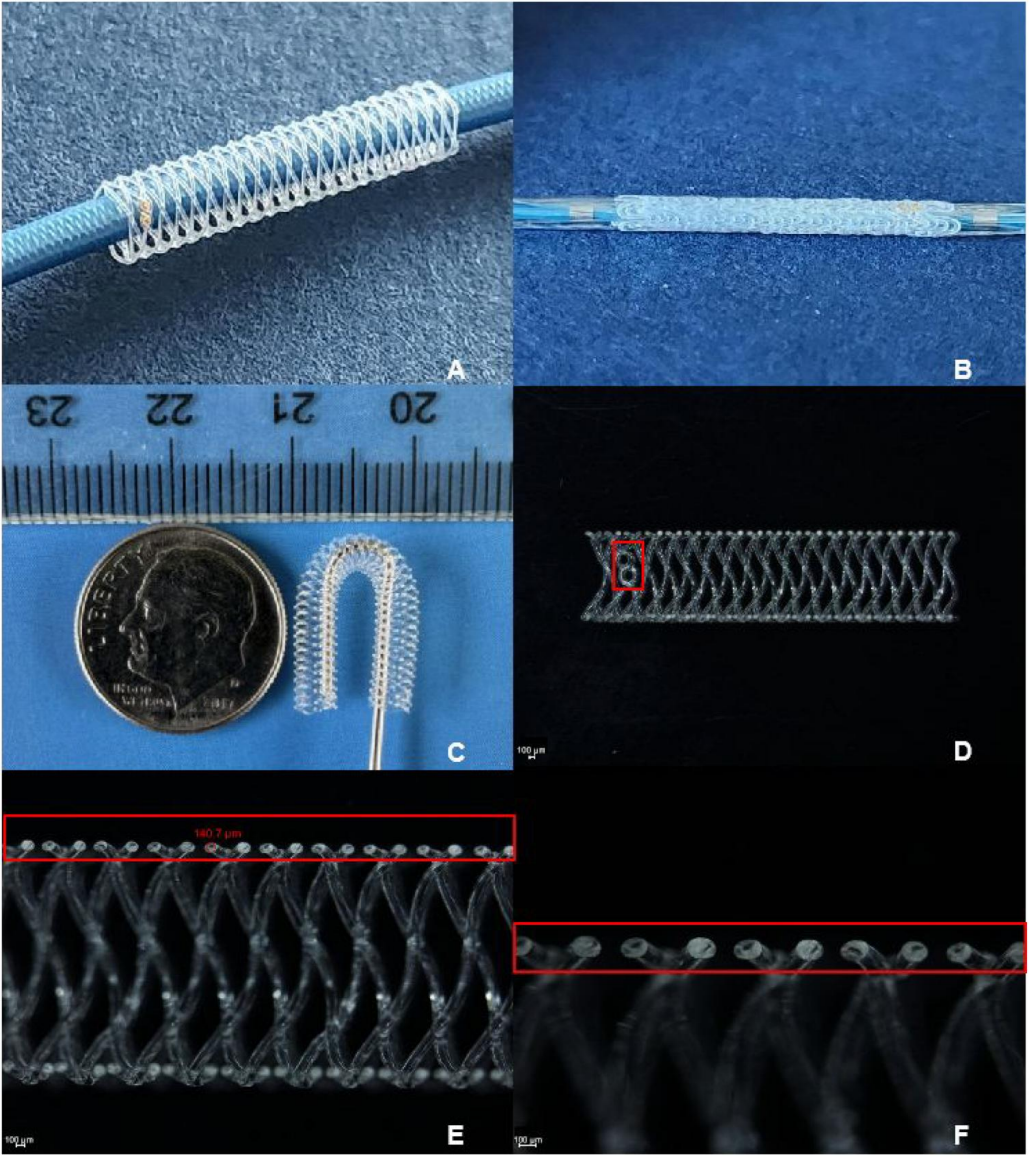
(**A**) 3D printed PLCL vascular stent (3.50 mm × 15 mm) with two gold markers on the left end of the stent; (**B**) Stent crimped onto a delivery balloon catheter; (**C**) Super-flexible structure of stent (3.00 mm × 33 mm); (**D**) Part of the longitudinal-sectional view of the stent (ID 3.0 mm), two printed holes for gold marker embedding can be seen on the left end of the stent (box); (**E**) Round cross sections of struts with an average diameter about 140 μm; (**F**) High magnification of the round cross sections of the stent struts.

The stents were fabricated from poly(L-lactide-co-caprolactone) 95/5 (M_w_ ≈ 65 K, Purac Asia Pacific Pte Ltd, medical grade), using a proprietary method of 3D printing with a rotating platform [29]. The PLCL pellets were heated and melt extruded at a temperature of 220°C through a 150 μm diameter nozzle which was mounted on a XYZ 3D printer. To characterize the mechanical properties of the extruded fibers of both PLCL and PLLA [Evonik Specialty Chemicals (Shanghai) Co., Ltd)], the fibers were collected directly after being extruded through the nozzle. To fabricate the stents, the extruded PLCL fibers were deposited onto the surface of a rotating mandrel driven by a serve motor to form stents. The stent structure consisted of helically interlaced polymeric fibers with a mean diameter of 140 μm, as shown in Figure 1. To visualize the stent under X-ray in clinical practice, two pairs of gold markers (99.9% purity) were embedded (Figure 1A) on both ends of the stent.

A drug-eluting coating of paclitaxel (Wuxi Taxus Pharmaceutical Co., Ltd) and PDLLA [Evonik Specialty Chemicals (Shanghai) Co., Ltd] mixture (at a ratio of 1:7) was applied to the surfaces of the stent at the abluminal side. Using an ultrasonic coating machine (Sonotech, DES2000), stents with 3 drug doses, i.e. 0.35 μg/mm^2^, 0.57 μg/mm^2^ and 2.28 μg/mm^2^ were prepared. Several stent sizes were prepared for the study.

### Fiber tensile tests

Fiber Tensile tests were performed using a Universal Tensile Testing Machine (Shanghai Fa Rui Instrument, Model FR-108C) (*n* = 10). The diameters of fibers were measured with a Laser micrometer (Keyence Corporation, LS-7601) before the test. The distance between the fixtures was 20 mm and the crosshead moving speed was set to 20 mm/min, in accordance with GB/T 14337-2022, Man-made fiber—Test method for tensile properties of staple fiber.

### Foreshortening of stents

Each stent (3.75 mm × 50 mm) was first crimped onto a balloon catheter in the stent crimping machine (Blockwise Engineering CX), and then, dilatated to its nominal diameter (3.75 mm) at 12 atm (1 215 900 Pa) pressure using an in-deflator in a water bath at 37°C. The test was performed according to ASTM F2081-06 (*n* = 10). The initial length of the crimped stent (*L*_0_) and the length of stent expanded to the nominal diameter (*L*) were measured with a stainless steel ruler (accuracy of 0.5 mm), and the foreshortening of stent *η* was calculated using the formula below.


$$\eta =\left|\frac{L_{0}-L}{L_{0}}\right|\times 100\%.$$

### Crystallinity

The Differential Scanning Calorimeter (DSC) was used to test the crystallinity of stents (*n* = 10, 3.75 mm × 50 mm), which was balanced at 10°C and ramped 10°C/min to 220°C. The crystallinity of stents was calculated by the following formula:


$$\mathrm{Crystallinity}=\frac{\Delta Hf}{\Delta Hm\infty },$$

where 100% crystallization melting enthalpy Δ*Hm*_∞_= 95 J/g [22], Δ*H_f_* represents the difference between the exothermic peak area and the endothermic peak area.

### *In vitro* degradation study

Each stent (2.75 mm × 12 mm) was placed into a test tube with 4 mL of phosphate-buffered solution (PBS, pH 7.2-7.4, formulated with phosphate buffer saline tablets purchased from Solarbio, Cat#P1000, Lot.No. 420G0329) and placed in an incubator shaker at 37°C for testing periods of 0, 15, 30, 60, 90 and 180 days. PBS was refreshed every 7 days, and the pH value was maintained at 7.4 ± 0.2. At each time point, the stents were taken out of the test tubes, rinsed with distilled water, dried in air at room temperature for 24 h and later used for the analyses of molecular weight, radial strength, surface morphology, weight loss and surface morphology.

#### Molecular weight measurement

Molecular weight of the stents was measured using a gel permeation chromatography (GPC, Waters 515) equipped with 7.80 mm × 300 mm Styragel HR 2 THF, Styragel HR 4 THF and Styragel HR 5 THF Columns at 35°C (*n* = 5). Chloroform was used as the mobile phase with a flow rate of 1 mL/min. The standard curve was created by using polystyrene as the reference standard material. About 10 mg of test sample was dissolved in 1 mL of chloroform for 24 h. After being filtered through a 0.22 μm microporous filter, the solution was transferred into a clean injection vial in the automatic sampling tray for GPC measurement. Each vial contained 20 μL solution.

#### Radial strength test

Radial strength of stents was measured using a radial strength Tester [Blockwise, Model TTR2 with 100 ibf (about 444.82 N) Load Cell] according to ASTM F3067-14, Guide for Radial Loading of Balloon Expandable and Self-Expanding Vascular Stents (*n* = 5). The lengths of the stents were measured before they were placed into the test chamber (preheated to 37°C) of the tester. The stent was then radially compressed to 40% of its initial diameter at a compression rate of 0.5 mm/s to measure its resistance to radial compression (in mmHg).

#### Weight loss

Prior to the initiation of the *in vitro* degradation and at designated degradation time points, the stents were weighed using an analytical balance (Mettler-Toledo AL104, Range: 0.1 mg–110 g, Accuracy: ±0.1 mg; *n* = 3).

#### Drug dose study

The purpose of this animal experiment was to study the effect of drug doses on the neointimal formation after stent implantation. A total of 9 British white pigs with an average weight of 37.46 kg were divided into 3 equal sized groups. Each group was implanted with stents (3.00 mm × 18 mm or 3.50 mm × 18 mm) with one of the three drug doses, 0.35 μg/mm^2^, 0.57 μg/mm^2^ and 2.28 μg/mm^2^. This experiment was conducted at Gateway Medical Innovation Center (Beijing) R&D Co., Ltd The ethical approval number (IACUC) is BJ-2021-02001.

The experimental animals were given 75 mg of clopidogrel and 100 mg of aspirin orally 72 h before the PCI procedures; The anesthesia was induced by intramuscular injection of Zoletil and inhaled isoflurane by respiratory intubation; Low-molecular-weight heparin 150 U/kg was administrated intravenously before catheter insertion; coronary artery access was established through the carotid artery. The left anterior descending (LAD) coronary artery, left circumflex branch (LCX) and right coronary arteries (RCA) of each pig were used for implantation of stents. Catheters of 6F (Medtronic, Inc.) and 0.014 inch guide wires (Terumo Corporation) were used. Angiography (GE Optima IGS 330) was conducted and the segments of the vessel with the right sizes were chosen for stent implantation with the balloon to artery ratios (BAR) of 1.0–1.1 in diameter. Both angiography and Optical Coherence Tomography (OCT, Lightlab C7, St Jude) were performed immediately after stent implantation. After implantation, 75 mg of clopidogrel and 100 mg of aspirin were given orally to each animal every day. One month after implantation, the animals were euthanized after angiography and OCT imaging were performed, and the vessels with stents were harvested for histopathology study. Stenosis was calculated based on the following equation:


$$\mathrm{Vascular}\;\mathrm{stenosis}\;\mathrm{rate}\;(\%)=\frac{MLD_{0}-MLD_{t}}{MLD_{0}}\;\times \;100\%,$$

where MLD_0_ is the minimum lumen diameter measured by angiography immediately after stent implantation (in mm), MLD_*t*_ is the minimum lumen diameter measured at time *t* after stent implantation (in mm).

#### Six-month animal implantation study

Stents with drug dose of 0.57 μg/mm^2^ were chosen for this study. Using the same PCI procedures as described above, a total of 4 stents (3.00 mm × 18 mm or 3.50 mm × 18 mm) were implanted in three animals (Table 1). The ethical approval number for this experiment is the same as above. Animals were euthanized at 6 months postimplantation, and the vessels with stent samples were explanted and fixed in neutral buffered Formalin and used for H&E staining and histopathology analysis.

**Table 1.** Summary of stent implantation for 6 months

Pig number	Location of implantation	Size	Duration
1	LAD and RCA	3.50 mm × 18 mm	6 months
2	RCA	3.50 mm × 18 mm	6 months
3	RCA	3.00 mm × 18 mm	3 months

Each stent was divided into proximal, middle and distal segments for evaluation. The histopathology slides were evaluated and photographed with a fully automatic fluorescence scanning microscope [Emmett (Xiamen) Technology Co., Ltd Model: Austar 43]. The scores of inflammation, injury and endothelialization were given according to the scales shown in Table 2 [38].

**Table 2.** Scoring system for histological evaluation of stent implant

Evaluating indicator	Score	Description
Endothelialization	1	less than 25% of the lumen is covered by endothelial cells
	2	25–75% of the lumen is covered by endothelial cells
	3	percentage of the lumen along circumference covered by in arterial lumen along the circumference higher than 75% of the lumen is covered by endothelial cells
Inflammatory reaction	0	none
	1	mild
	2	moderate
	3	marked
	4	granulomatous inflammation: Granuloma observed around more than 25% stent struts
Vascular injury score	0	Intact internal elastic plate
	1	broken internal elastic plate
	2	fractured internal elastic plate and middle membrane
	3	broken outer elastic plate

The explanted stent samples were used to measure their molecular weight changes to study the degradation process of the stents. After lyophilized for at least 8 h, the dried stent samples were put into 1 mL chloroform at a solid to liquid ratio of 10 mg/mL for at least 12 h to dissolve the stent. A GPC (Waters 515) was used to perform the molecular weight measurement.

## Results

The stents fabricated with the 3D printing with a rotating platform system showed smooth surfaces. The gold markers can be visualized as shown in Figure 1A. Stents could be crimped tightly onto balloon catheters using a stent crimper (Figure 1B). The stent was kink resistant and super flexible as shown in Figure 1C. The minimum diameter when the stent bended 180° without kinking was about 3 mm for a 3-mm diameter stent. Also, the cross sections of the stent struts were round (Figure 1D–F).

### Tensile test of extruded fibers

**Figure 2.**
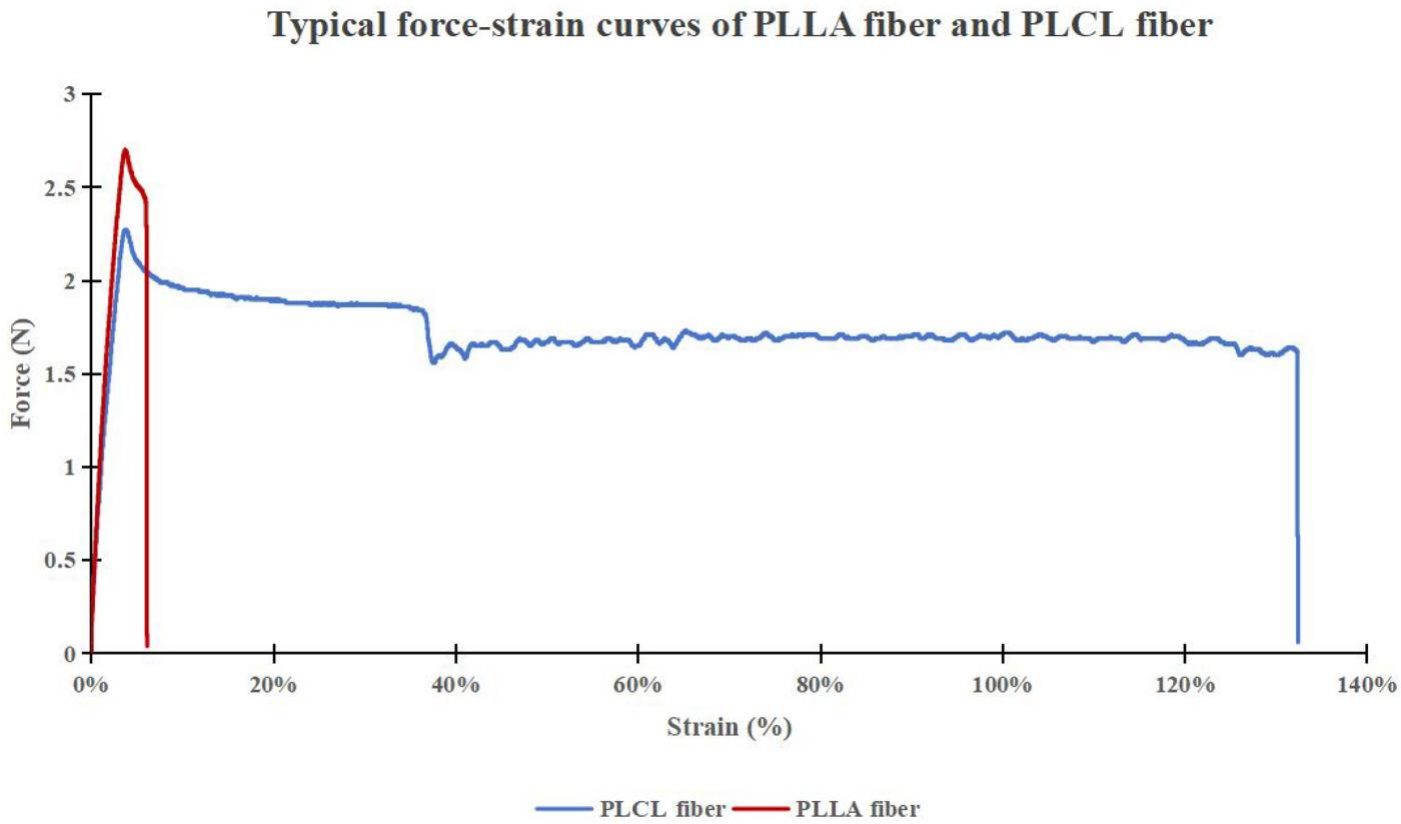
Typical force–strain curves of PLLA fiber and PLCL fiber.

As a comparison, tensile tests were performed on both PLCL and PLLA fibers. Results showed that PLCL fibers exhibited a far superior elongation at break yet a similar Young’s modulus as compared to that of PLLA fibers (Figure 2 and Table 3). Specifically, PLLA fibers exhibited an elongation at break of merely 8 ± 2% (*n* = 10) while PLCL fibers showed a much greater elongation at break of 289 ± 141% (*n* = 10).

**Table 3.** Tensile properties of PLLA and PLCL fibers

Material	Diameter (mm)	Young’s modulus ± SD (MPa)	Yield strength ± SD (MPa)	Breaking strength ± SD (MPa)	Elongation at break ± SD (%)
PLLA	0.230 ± 0.004	2017.13 ± 176.48	59.21 ± 3.42	52.73 ± 3.62	8 ±2
PLCL	0.230 ± 0.032	1943.08 ± 134.77	51.17 ± 2.39	42.61 ± 3.97	289 ± 141

### Foreshortening of the stents

As shown in Table 4, the PLCL stents with the patented closed cell structure design showed a low foreshortening of 3.57 ± 0.72% (*n* = 10).

**Table 4.** Properties of PLCL stents.

	Foreshortening rate（%）	Crystallinity（%）
Max	4.66	35
Min	2.07	31
Average (*n* = 10)	3.57	33
Standard deviation	0.72	1

### Crystallinity

**Figure 3.**
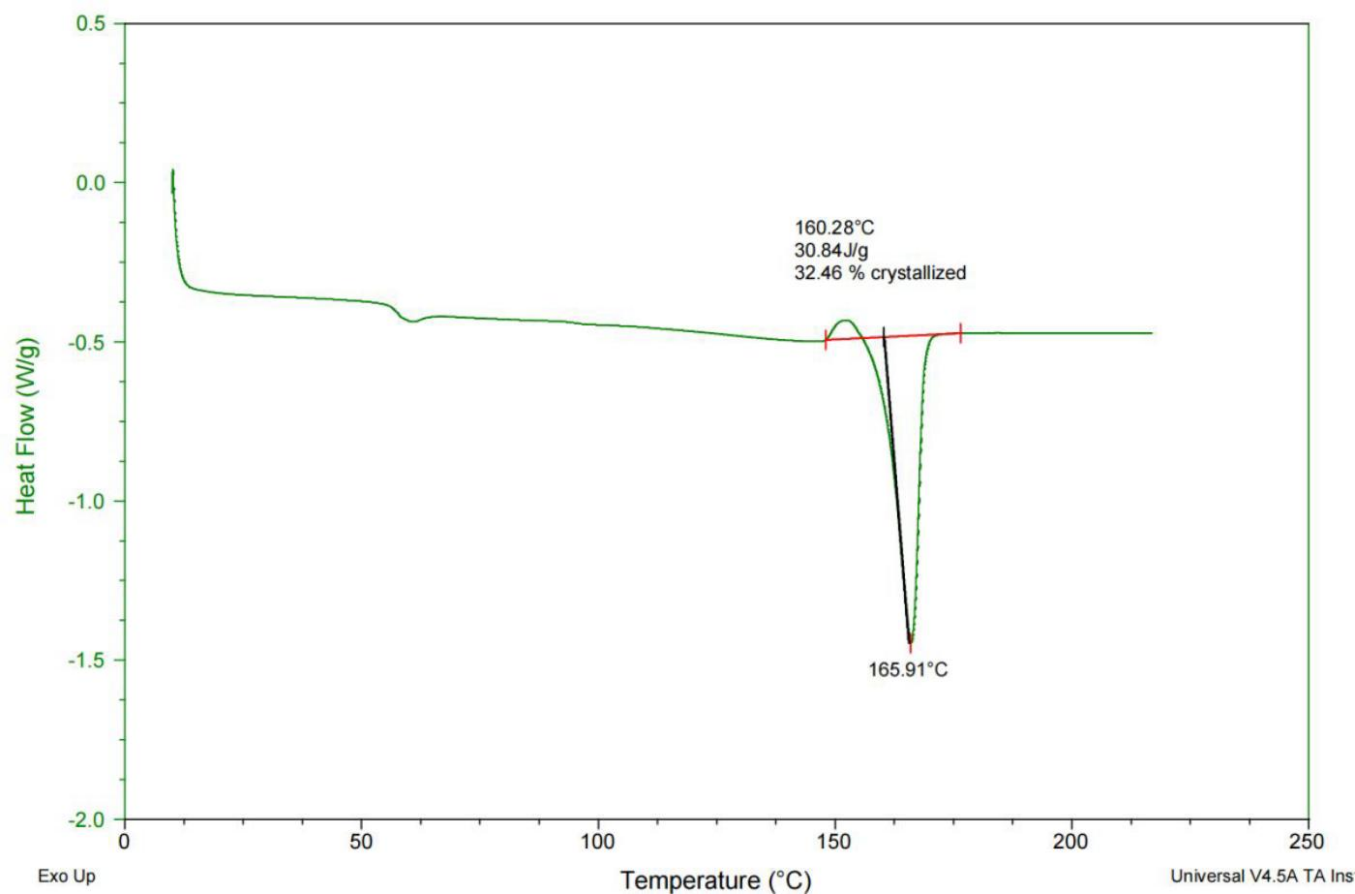
Typical DSC curve of PLCL stent.

A typical crystallinity curve of PLCL stent was shown in Figure 3 and the crystallinity of PLCL stents was 33 ± 1% (*n* = 10, Table 4).

### *In vitro* degradation

**Figure 4.**
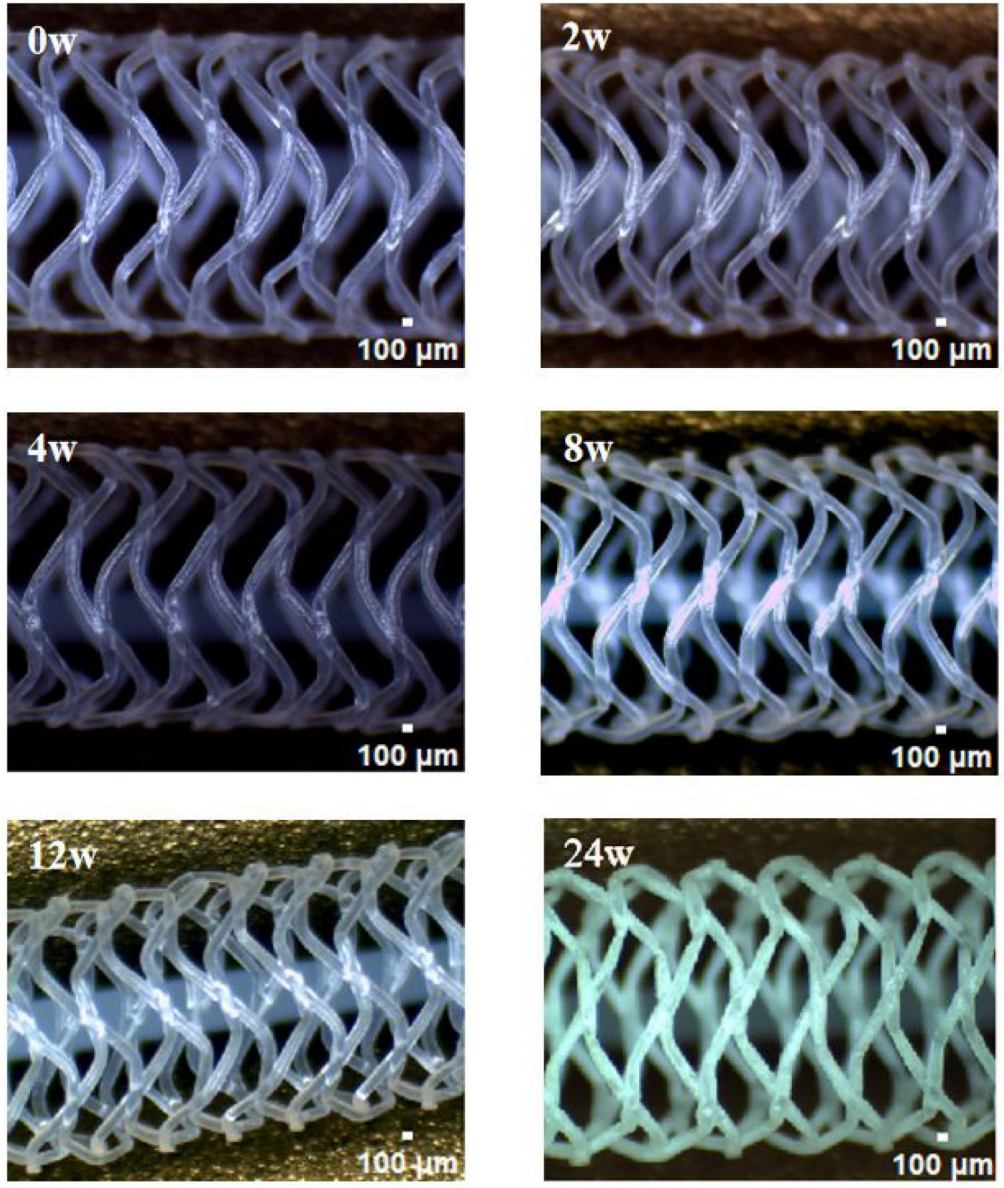
The morphological changes of the PLCL stents during the *in vitro* degradation study.

During the degradation study, the stent struts gradually changed with from translucent to opaque, with no structural changes or break down of the struts were observed during the entire 180 days of study (Figure 4).

#### Molecular weight

**Figure 5.**
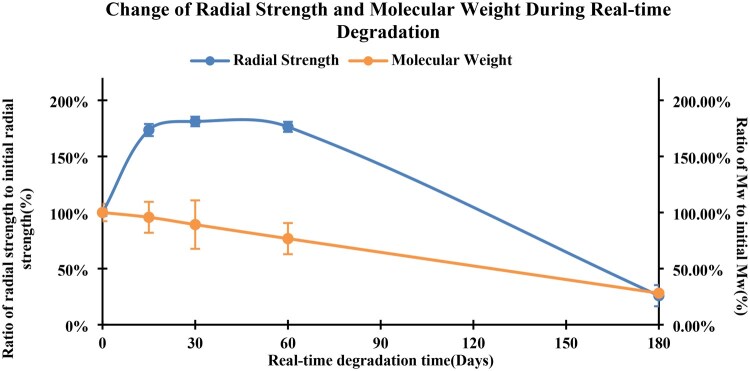
Real-time degradation of vascular stents with changes in molecular weight and radial strength.

Figure 5 showed that the molecular weight (weight average) changed over time (15, 30, 60 and 180 days) of the PLCL stents *in vitro*. The data indicated that the molecular weight started to decrease after the start of degradation study and retained 28% of the original molecular weight (143 319  ±  4282 Da) at the end of the study.

#### Radial strength

Figure 5 also showed the radial strength change of PLCL stents during the real-time degradation study. With the real-time degradation process going on, the radial strength of the stents increased gradually within the first month and then started to decrease after the second month.

It was observed that although the molecular weight of the stents started to decrease, the radial strength of PLCL stents increased dramatically and reached 1182 ± 141 mmHg after being immersed in PBS within the first month after the test started, which corresponded to the critical reendothelialization period of the stents. The possible reasons for this phenomenon could be that when the PLCL copolymer initially absorbed water, the rapid expansion of the polymer matrix might cause an increase in internal stress, thereby enhancing the radial strength within a short period of time [39]. The interface between PLLA and PCL in PLCL played a crucial role in the mechanical properties of the copolymer. The hydration effect might alter the interactions at the interface, leading to stress concentration and an increase in radial strength [40]. Although initial water absorption might temporarily increase the radial strength, in the long run, hydrolysis degradation would cause the polymer matrix to soften and the mechanical properties to decline. After 3 months of degradation *in vitro*, although the radial strength of the stents had gradually decreased, it was still higher than the initial radial strength value of 652 mmHg. At the end of 6 months of the in *vitro* degradation, the radial strength of the stents decreased to 169 mmHg which was higher than normal blood pressure. These data indicated that the PLCL stents could provide enough radial support for 3–6 months to allow the vessel remodeling to complete.

#### Weight loss

As shown in Table 5, during six months of *in vitro* real-time degradation, the weight of the stents did not decrease significantly.

**Table 5.** Weight loss during real-time degradation (*n* = 3).

Degradation time (Days)	Original weight ± SD (mg)	Weight ± SD (mg) after degradation	Percent change (%)
0	20.17	/	100.00
30	17.20	17.13	99.59
60	20.97	20.87	99.52
180	16.87	16.27	96.44

### Drug dose study

The experimental results are shown in Table 6 below. Animals in the first group (drug does of 0.35 μg/mm^2^) showed severe in-stent stenosis, and one of the stent showed complete stent occlusion with no blood flow. No apparent stenosis was observed in the second group of blood vessels with stents (drug dose of 0.57 μg/mm^2^). The animals in the third group (drug dose of 2.28 μg/mm^2^) had one death at day 13. Autopsy showed that the death was caused by acute in-stent thrombosis. Further examination on the explants showed that stents in group 3 had poor endothelialization after a month of implantation. Based on the above findings, stents with drug dose of 0.57 μg/mm^2^ were used for further animal study.

**Table 6.** Animal experiment results of screening dose.

Groups	Drug dose （μg/mm^2^）	Percent stenosis （%）	Number of vessel occlusions	Observations
1	0.35	56.77 ± 40.21	1	Neointimal hyperplasia with significant stenosis in the lumen of the stent
2	0.57	0.00 ± 0.00	0	The stent struts completely covered by a thin neointimal layer; and the lumen patent without any notable narrowing
3	2.28	66.67 ± 57.74	2	Uncovered stent struts with poor endothelialization after a month of implantation; one death caused by acute in-stent thrombosis

**Figure 6.**
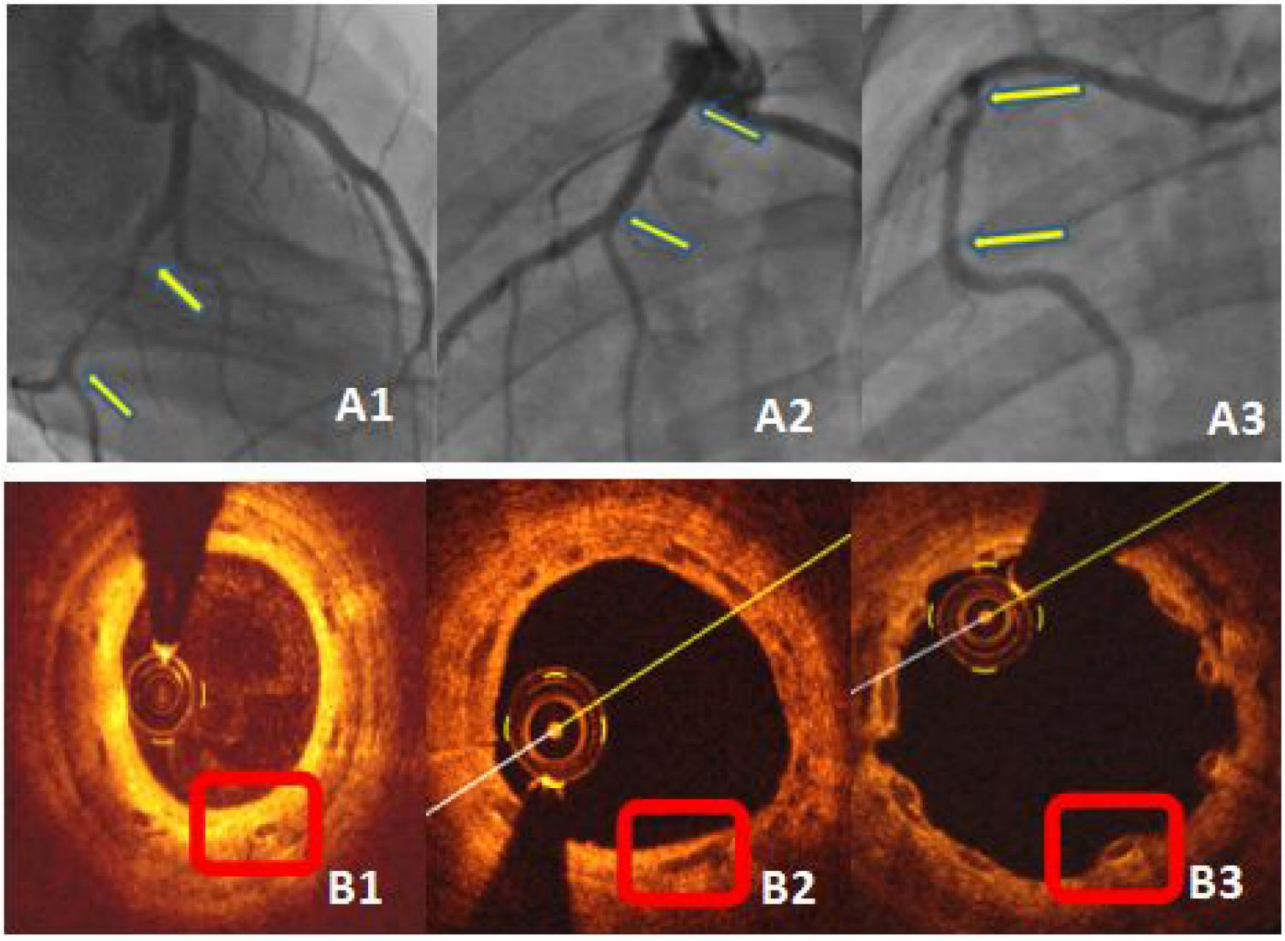
Representative angiographic and OCT images of the blood vessels one month after implantation of stents with different drug doses. As can be seen from the (**A1, B1**), in the first group, the neointima is relatively thick at 1 month, and the stent segment of the blood vessels showed stenosis. As can be seen from the (**A2, B2**), in the second group, endothelization was completed with moderate neointima thickness and no stenosis in the stent segment in the angiography image. As can be seen from the (**A3, B3**), in the third group, the endothelization of the stent was incomplete.

As seen from Figure 6, after implantation of stents with different doses of drug coating, the drug doses greatly affected the extent of the neointima forming over the struts of the stents. Among the three does groups, the neointimal formation was the greatest in the 0.35 μg/mm group resulting significant stenosis and neointimal hyperplasia (Supplementary Figures A1 and B1), while only moderate in the 0.57 μg/mm^2^ group with no stenosis and optimal strut coverage by neointima (Supplementary Figures A2 and B2), but very little in the 2.28 μg/mm^2^ group with little or no strut coverage by neointima (Supplementary Figure B3). It was found that with the increase of the drug dose to 2.28 μg/mm^2^, the stent struts were uncovered by the neointima after 1 month implantation and exposed to blood flow, suggesting the drug dose of 2.28 μg/mm^2^ was too high and fully inhibited the neointimal formation with a risk in thrombosis. Therefore, the stents with a paclitaxel coating drug dosage at 0.57 μg/mm^2^ was both effective and appropriate in terms of stent healing and inhibiting stent restenosis.

### Six-month animal implantation study

As can be seen from Table 7, the diameter of coronary arteries in the selected implantation segment ranged from 2.9 mm to 3.6 mm. At the first month of postimplantation, the stenosis rate was 24.44 ± 8.05%, and it maintained at this level until the fifth month. After that, the stenosis started to decrease and reached 7.13 ± 12.38% at 6 months. Overall, the stents with paclitaxel coating at a drug concentration of 0.57 μg/mm^2^ showed a low stenosis during the first 6-month implantation in the porcine model.

**Table 7.** Minimum lumen and stenosis rate at each time-point.

Vascular stent number	Implantation location	Sizes	Immediate	One month		Two months		Three months		Four months		Five months		Six months	
			MLD (mm)	MLD (mm)	Percent Stenosis (%)	MLD (mm)	Percent Stenosis (%)	MLD (mm)	Percent Stenosis (%)	MLD (mm)	Percent Stenosis (%)	MLD (mm)	Percent Stenosis (%)	MLD (mm)	Percent Stenosis (%)
1	LAD	3.50 mm × 18 mm	2.80	1.80	35.70	2.09	25.30	1.90	32.14	1.60	42.85	2.00	28.57	2.20	21.43
2	RCA	3.50 mm × 18 mm	2.70	2.50	21.87	2.80	0.00	2.90	0.00	2.30	14.81	2.30	14.81	2.80	0.00
3	RCA	3.50 mm × 18 mm	3.40	2.60	23.52	2.40	29.41	2.70	20.59	2.70	20.59	3.00	11.76	3.40	0.00
4	RCA	3.00 mm × 18 mm	2.40	2.00	16.67	1.60	33.33	1.80	25.00	NA	NA	NA	NA	NA	NA
***Average***			2.83	2.23	24.44	2.22	22.00	2.33	19.42	2.20	26.08	2.43	18.38	2.80	7.13
***Standard deviation***			0.42	0.39	8.05	0.51	15.05	0.56	13.83	0.56	14.81	0.51	8.96	0.60	12.38

Note: MLD: minimum lumen diameter.

**Figure 7.**
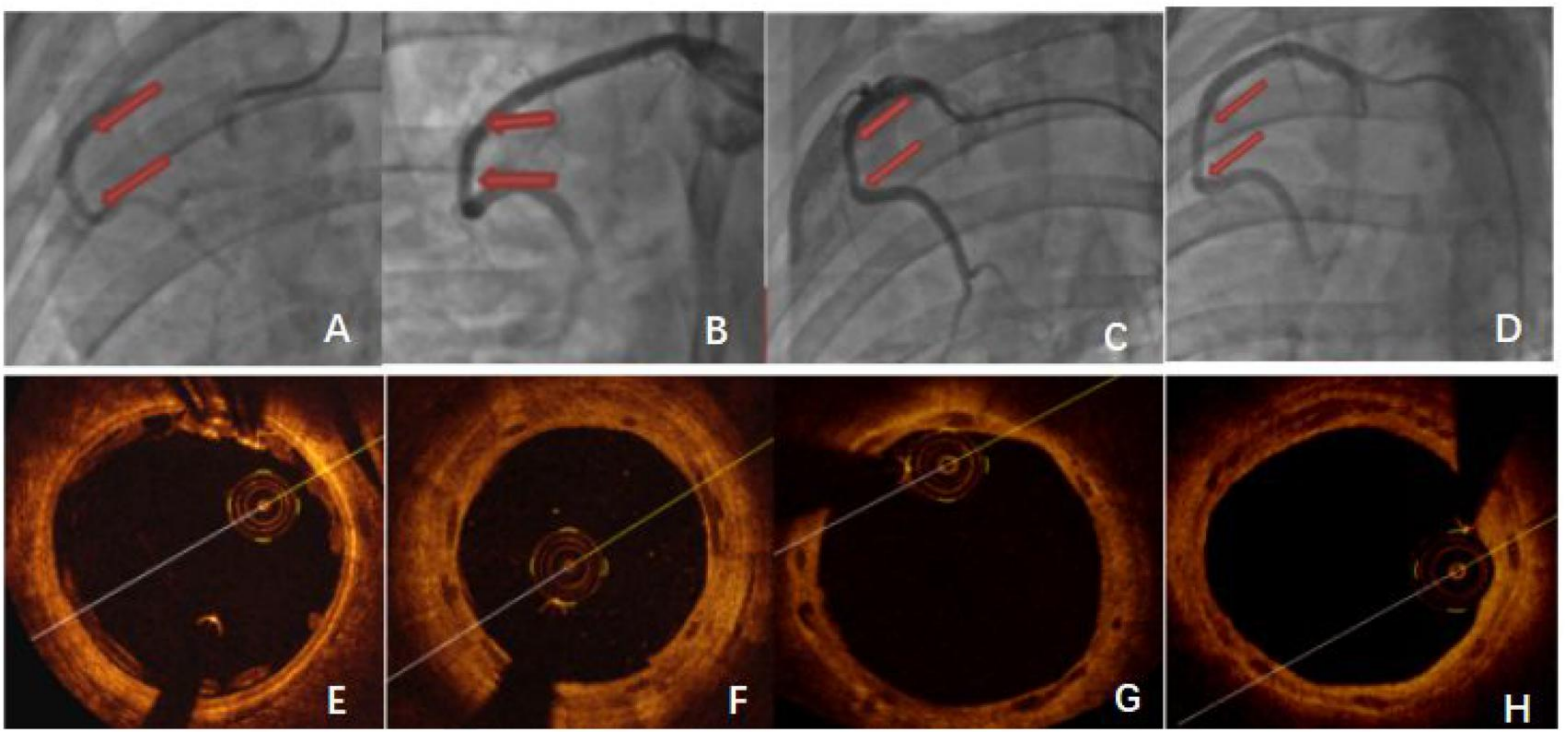
The representative QCA and OCT images. (**A**, **E**) represent QCA and OCT images immediately after stent implantation, (**B**, **F**) represent 1-month follow-up images, (**C**, **G**) represent images at 3-month follow-up, (**D**, **H**) represent 6-month images after stent implantation.

As can be seen from Figure 7, immediately after stent implantation, there was no stenosis in the angiographic stent segment. OCT showed that the stents were completely endothelialized one month after implantation, and without obvious stenosis at 3 and 6 months.

**Figure 8.**
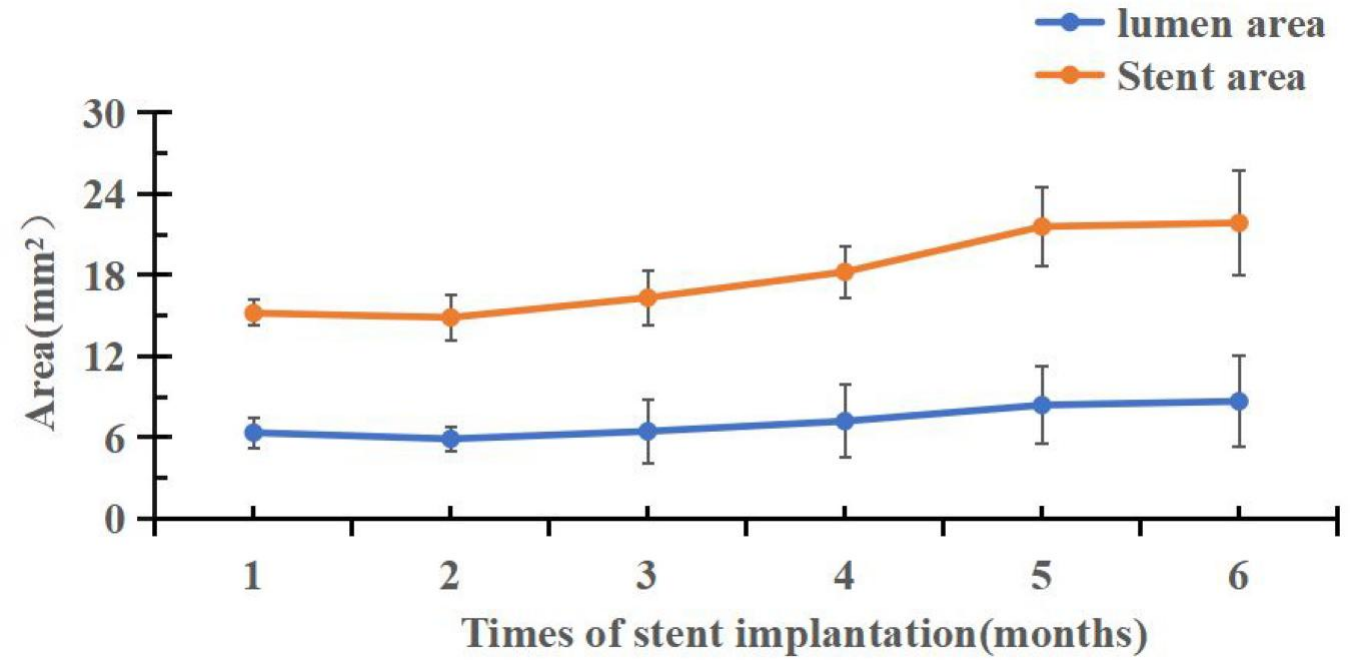
Changes in vascular lumen area and stent area.

As can be seen from Figure 8, OCT data showed that the area of neointima (The area of the outer surface of the stent minus the area of the vascular lumen) gradually increased from 1 month of implantation to 5 months of implantation and decreased from the 5 months to the 6 months. At the 5 months, the maximum neointimal area was 4.82 mm^2^ ± 0.53 mm^2^.

**Figure 9.**
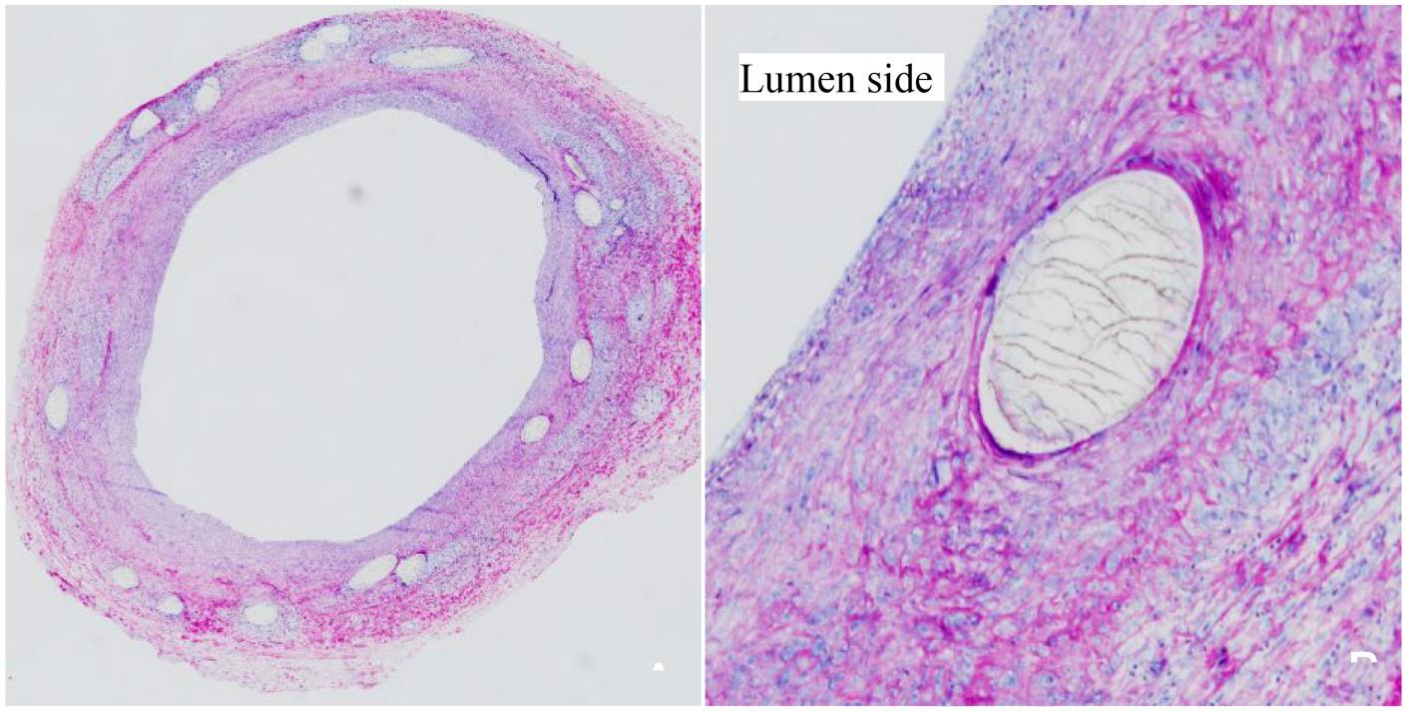
Typical pathological images with H&E staining. As can be seen from (**A**), the vascular endothelization of the stent segment with complete neointima coverage. As shown in (**B**), there is a stent covered with newly formed inner membrane. Cracks were observed throughout the struts of stent, indicating that struts have begun to disintegrate due to degradation.

As shown in Table 8, during the 6-month study, the stents showed complete endothelialization, with an injury score of 2.25 ± 0.87 and an inflammation score of 1.67 ± 1.23 at 6-month, respectively. It can be seen that inflammation continued to exist for up to 6-month. According to the data of neointima area measured by OCT, the area of neointima at 6-month is lower than that at 5 months, therefore, it is likely that the inflammation score would continue to decrease after 6 months. The scoring reference standards are shown in Table 2 [38]. The results of pathological tissue sections of the stent over a 6-month study, completely encapsulated within the blood vessel, are shown in Figure 9. It can be seen that although the stent rods have developed cracks, they have not yet been stained.

**Table 8.** Pathological scores at 6-month implantation (*n* = 3).

	Inflammation score	Injure score	Endothelialization score
Six-month after stent implantation	1.67 ± 1.23	2.25 ± 0.87	3.00 ± 0.00

The experimental animals were euthanized at 6-month after implantation, and a stent segment was taken for molecular weight measurement. The weight average molecular weight (*M*w) value was about 29.7 kDa. It showed that the PLCL stents degraded with time after being implanted *in vivo*, and the molecular weight retained about 18.37% of its original value at the end of 6-month implantation.

## Discussion

After the withdrawal of the Abbott’s ABSORB^®^ BVS stents from the market, a next generation of bioresorbable stents is needed to meet the clinical needs. The ideal bioresorbable stents, as learnt from ABSORB^®^ BVS stents, should have thinner struts, faster degradation rate and cause no thrombosis after implantation before they are completely absorbed in the body. Additionally, the polymer should have sufficient toughness so that the stents made from it can withstand the large deformations during the stent crimping and balloon dilatation processes without causing strut cracking or breaking; in addition, the stents should maintain sufficient radial strength for at least 3–6 months before stent structure disintegration so that the vessel could be completely remodelled, thereby eliminating the risk of late or very late stent thrombosis.

To develop such kind of ideal stent, we have come up with a new bioresorbable stent which was 3D printed with a new stent structure design and PLCL material. The struts of the stents showed very smooth surfaces (Figure 1A) with round cross sections (Figure 1D). The thickness of the struts was about 140 µm which was thinner than that of ABSORB^®^ BVS (∼160 µm). It was believed the struts with round-cross sections could be easily embedded into vessel walls after high-pressure balloon dilation so that the endothelialization process could be accelerated and the late and the very late thrombosis could be prevented.

For better identification of the radiolucent bioresorbable polymer stents during the PCI procedures, 2 pairs of gold markers were inserted into the printed marker holes at both ends of the stents (Figure 1A). With the novel super-flexible close-cell structure design (Figure 1C), the stents could be tightly crimped onto balloon catheters (Figure 1B) and showed a very low foreshortening of less than 5% (Table 4). The low foreshortening of stent suggests that the stents can be delivered and dilated to cover the lesion precisely. The stent showed excellent flexibility and antikinking behavior as shown in Figure 1C. Good flexibility or antikinking properties are important to vascular stents after being implanted into vessels because stents must resist not only the radial load caused by the vessel wall and the blood pressure but also additional mechanical loads from the surrounding tissue caused by the physiology movements, such as bending and stretching. Coronary stents, when implanted into the right coronary artery (RCA), are stressed because of the excessive movement of the RCA and repetitive kinking during the cardiac cycle. To reduce the risk of in-stent restenosis caused by a stent indicated lumen loss of the vessel, the antikinking behavior of stents is of interests for clinicians, stent manufacturers and regulatory agencies.

Previous studies [22] have shown that a PLCL copolymer with a caprolactone ratio of 5% exhibits enhanced flexibility and faster degradation. In this study, the extruded fibers from the 5% PLCL copolymer showed much higher elongation at break than that of PLLA fibers but had similar elastic modulus to that of PLLA fibers (Figure 2 and Table 3). The stents showed almost completed crystallization because the crystallization peak was quite small in the DSC curve (Figure 3). The newly patented close-cell stent design [32] was adopted so that the thickness of the struts could be reduced while the stents still maintained sufficient radial strength to support the vessel and prevent stent recoil. In addition, the 3D printing with a rotating platform process [22] could produce stents in a very cost-efficient way, for example, a PLCL coronary stent could be printed in less than 1 min. Also, the cross-sections of the stent struts are round (as shown in 1D), which makes the struts easily be embedded into vessel walls during stent deployment process so that the stent endothelization process can be accelerated.

The 3D printed PLCL stents with the patented closed cell structure design showed a low foreshortening of 3.57 ± 0.72%. Foreshortening is one of the important characteristics of a balloon expandable stents. A low foreshortening means that the stents can be delivered and dilatated to cover the lesion more precisely. For a metallic balloon expandable stent, a foreshortening of less than 10% is preferred.

As the degradation study went on, the stent struts gradually changed from translucent to opaque, with no structural changes or break down of the struts were observed during the entire 180 days of study (Figure 4). The molecular weight changes over time indicated that the PLCL stents had a faster degradation behavior in the *in vitro* degradation study (Figure 5) as compared to the degradation profile of PLLA. In our previous study for a 3D printed PLLA stent, PLLA molecular weight decreased only 50% in a 7-month *in vitro* degradation study [29].

During six months of *in vitro* real-time degradation, the weight of the stents did not decrease significantly (Table 5). Studies have shown that the mass of the PLLA stent remained unchanged throughout the degradation test, but the PLCL stent showed a 5% loss in mass on the 90th day and an increase of up to 12% after 120 days [41]. This result bears some resemblance to the findings of this study. The molecular weight reductions without much weight changes may be caused by the following three reasons. First, chain scission occurs before dissolution: The situation where the molecular weight decreases but the weight changes little indicates that the copolymer mainly undergoes chain scission rather than dissolution or disintegration of the material. This means that ester bond hydrolysis leads to a decrease in the molecular weight of PLLA and/or PCL segments, but the low-molecular-weight fragments generated by scission remain within the material, thus resulting in no significant weight loss [42, 43]. Second, water absorption and degradation: water absorption is one of the initial steps of polymer degradation [42]. The water absorption capacity of PLLA and PCL copolymers is affected by their composition and morphology [44]. If the degradation products are water-soluble, they will eventually be released into the surrounding environment, resulting in weight loss. However, if the degradation products remain in the polymer matrix during the initial stage of degradation, only a decrease in molecular weight will be observed [45]. Third, The influence of crystallinity: The crystallinity of PLLA and PCL also affects the degradation rate. The amorphous regions are usually more prone to hydrolysis than the crystalline regions [46]. If the PLLA segments are degraded preferentially, resulting in a rapid decrease in molecular weight, while the PCL segments degrade more slowly and can still maintain the integrity of the material, the weight change may not be significant [47].

The novel 3D printed PLCL stent had a strut thickness of about 140 µm and showed a high radial strength of 652 mmHg thanks to the new stent structure design (Figure 5). Both *in vitro* and *in vivo* study showed that the radial strength of the stent was further increased by 1.8 times within the first month after being immersed in simulated body fluid (Figure 5). The radial strength maintained at this level for one more month and then started to decline. Figure 5 suggested that after 3 months of degradation *in vitro*, although the radial strength of the stents had gradually decreased from their peak values, it is still higher than the initial radial strength of 652 mmHg. At the end of 6 months of the *in vitro* degradation, the radial strength of the stents decreased to 169 mmHg which was still higher than normal blood pressure. These data indicated that although PLCL material degrades faster that PLLA, PLCL stents could provide enough radial support for 3–6 months to allow the vessel remodeling to complete. The initial increase in radial strength may be due to the plasticizing effect of water that diffused into the polymer matrix.

With a faster degradation polymer PLCL, more degradation products could be produced and accumulated during the stent degradation process. As a result, excessive neointimal hyperplasia may occur. Therefore, it was suggested that a long-lasting drug coating or a slower drug release coating would be necessary for inhibition of initial inflammatory reactions and in-stent stenosis. Therefore, the selection and dosage of antiproliferative drugs need to be re-evaluated.

The majority coating of drug-eluting stents (DES) currently used in clinical practice are polymer-based, and they typically release either rapamycin (sirolimus) or paclitaxel (Taxol™) [48]. Both compounds have strong antiproliferative properties that hinder cell proliferation cycle progression. Sirolimus has been successfully used as an eluting drug from PLLA matrix stents and has shown satisfactory clinical results in terms of inhibiting in-stent restenosis. However, when a fast-degradation polymer is employed as a stent matrix, the use of sirolimus as the eluting drug may not be suitable due to the increased production of degradation products, which can lead to much severer tissue responses within the vessels.

In comparison, paclitaxel is a water-insoluble drug, resulting in a slower release from a coating into an aqueous environment. For faster-degradation stents with more degradation products accumulation locally, a paclitaxel/PDLLA coating with slower drug release may be a better choice. Several studies suggest that the drug release from a paclitaxel-coated DES platform was slower than that from a rapamycin-eluting DES platform [49, 50]. However, the use of paclitaxel in a drug-eluting coating on a biodegradable polymer coronary stent has not been reported.

The drug dose study with porcine model indicated that the drug dose of 0.35 μg/mm^2^ was low because the vessels showed a relatively thick neointimal formation (Figure 6). The drug dose of 2.28 μg/mm^2^ was so high that the struts were not fully covered by the neointima after 1 month implantation. Only the thickness of the neointima in the 0.57 μg/mm^2^ group was moderate. Therefore, the stents with a paclitaxel coating drug dosage at 0.57 μg/mm^2^ is both effective and appropriate in terms of inhibition of in stent restenosis.

*In vivo* stent implantation study showed that the stents with a dose of paclitaxel of 0.57 μg/mm^2^ was suitable for preventing intima hyperplasia in the stented segment of the vessel after implantation. Quantitative coronary angiography (QCA) data showed that the diameter of coronary arteries in the implanted stent segment ranged from 2.9 mm to 3.6 mm. At the first month of postimplantation, the stenosis rate was 24.44 ± 8.05%, and maintained at this level for 5 months. After that, the stenosis started to decrease and reached 7.13 ± 12.38% at 6 months. This result is no less significant than the 6-month outcome of our team’s previous study on a 3D-printed PLCL stent containing 20% nano-hydroxyapatite (with a stenosis rate of 14.5%) in pig coronary arteries [22]. Overall, the stents with paclitaxel coating at a drug concentration of 0.57 μg/mm^2^ showed a low stenosis during the first 6-month implantation in the porcine model. This is similar to the result achieved by Han *et al*. in their development of a high-precision PLCL stent (with a layer thickness of 50 μm) in rabbit carotid artery transplantation, where the 6-month patency rate was 90% [51].

OCT data showed that the area of neointima (The area of the outer surface of the stent minus the area of the vascular lumen) gradually increased from 1 month of implantation to 5 months of implantation and decreased from the 5 months to the 6 months (Figure 8). At the fifth month, the maximum neointimal area was 4.82 mm^2^ ± 0.53 mm^2^ which is lower than the clinically define stenosis (defined as 50% reduction in lumen diameter, which was calculated as 6.75 mm^2^ in this case). Because the thickness of the stent struts was 140 μm, the minimum area of neointima that was required to cover the surface of the struts was calculated as 1.42 mm^2^ that also contributed to the overall neointimal area. The OCT results were in agreement with the stenosis rate obtained from the above QCA study (Table 7 and Figure 7). This dose of 0.57 μg/mm^2^ is only 1/10 of that a drug coated balloon and is a safe dose. The combination of PLCL stent material and paclitaxel/PDLLA coating is suitable and warrants for further clinical studies.

During the 6-month animal implantation study, the stents showed quite low injury score of 2.25 ± 0.87 and an inflammation score of 1.67 ± 1.23 at 6 months, respectively. According to the data of neointimal area measured by OCT, the area of neointima at 6 months is lower than that at 5 months, therefore, it is likely that the inflammation score would continue to decrease after 6 months.

Animal models have certain limitations. The healing speed of blood vessels in pigs is 2–3 times faster than that in humans, which may require correction of the time scale when extrapolating the degradation data to the human body [52]. And this experiment only tested three dosages on 9 animals. The number of animals was relatively small, and no control group was set up. Moreover, the experimental period was only 6 months, and the pathological section results were only available for 6 months. It was impossible to trace the changes such as endothelialization, inflammation and damage. No immunohistochemistry was conducted to reflect the changes of endothelial cells and smooth muscle cells. Therefore, further effort is needed to conduct more animal experiments to fully verify the degradation cycle and the safety of the material. Future research can also combine computational models such as finite element analysis to predict long-term performance [53].

## Conclusions

The newly developed 3D printed paclitaxel eluting PLCL stents with super flexible structure and round cross sectional struts showed a faster degradation profile, thinner struts and satisfactory biocompatibility. The stents exhibited excellent radial strength, delivery and strut apposition capabilities in animal studies. With a paclitaxel dose of 0.57 μg/mm^2^ in the paclitaxel/PDLLA coating, the drug eluting stents showed very low degree of stenosis within the first 6 months of implantation. Therefore, the newly developed 3D printed PLCL stent with paclitaxel as eluting drug is very promising candidate for next generation bioresorbable coronary stent.

## Supplementary Material

rbaf073_Supplementary_Data
