# Supplementary material for: A novel paclitaxel eluting bioresorbable vascular stent with a super flexible stent structure and round cross section struts fabricated using 3D printing technology with a rotating platform
Source: Regen Biomater. 2025 Jul 9;12:rbaf073. doi: 10.1093/rb/rbaf073 (PMC12371334; doi:10.1093/rb/rbaf073)
Supplement: rbaf073_Supplementary_Data [file rbaf073_supplementary_data.doc]

**supplementary material**

**1. FTIR**


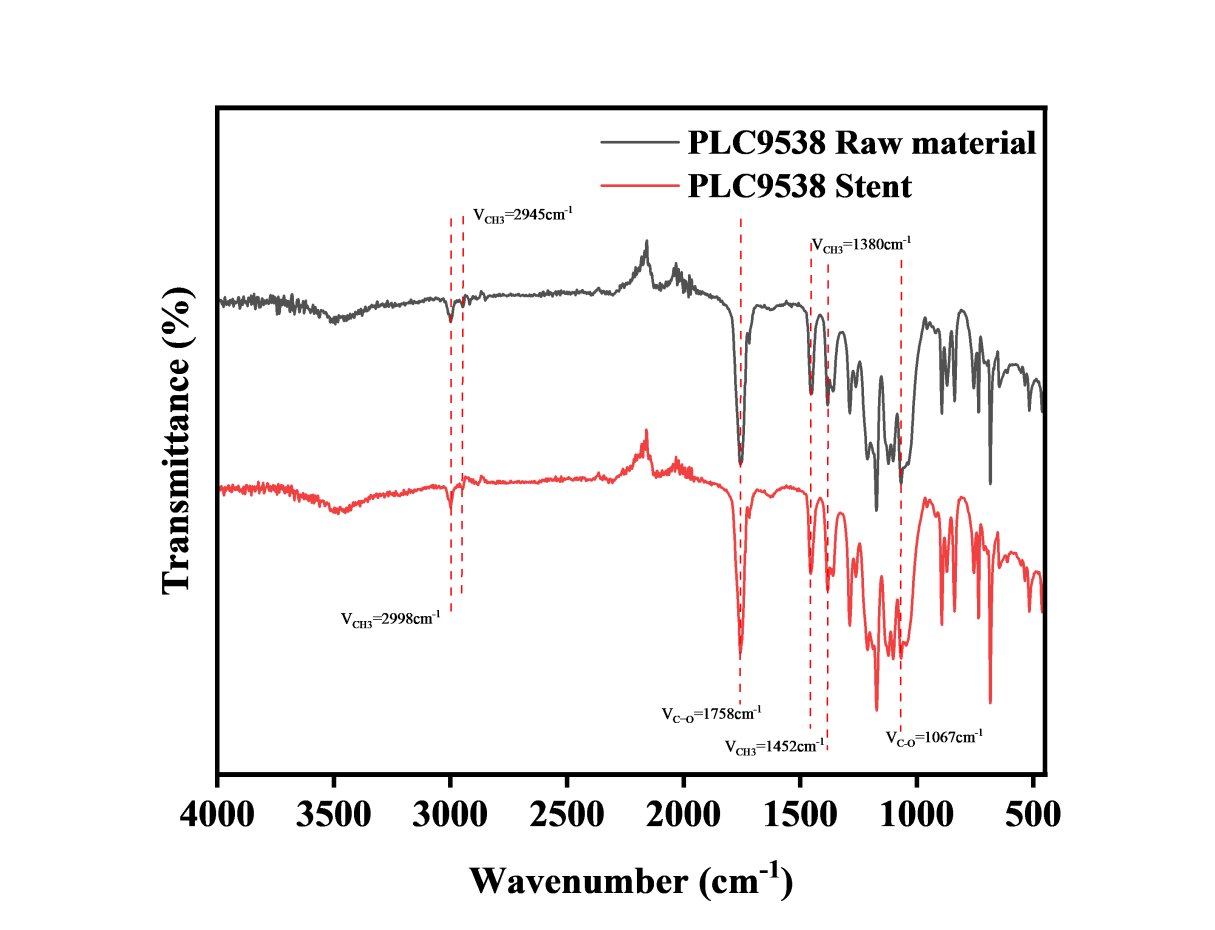


Figure 1. FTIR spectra of PLCL raw material and stent.

Figure 1 shows FTIR spectra of both PLCL raw material and the stent. In these two identical spectra, the characteristic stretching frequencies of C=O, -CH_3_ asymmetric, -CH_3_ symmetric and C-O are shown at positions of 1758, 2998, 2945 and 1067 cm^−1^, respectively, and the bending frequencies of -CH_3_ asymmetry and -CH_3_ symmetry are shown at 1452 and 1380 cm^−1^, respectively. This indicates that the PLCL polymer did not change during the printing process because there were no new bonds formed.

**2 *In* *Vitro* Degradation**

**2.1Molecular weight changes**

Table 1. Molecular weight changes during real-time degradation (n=5)

| **Degradation Time (Days)** | **Molecular Weight ± SD (Da)** | **Percentage (%)** |
| --- | --- | --- |
| 0 | 143319 ± 4282 | 100% |
| 15 | 137215 ± 7732 | 96% |
| 30 | 127891 ± 6126 | 89% |
| 60 | 109970 ± 6446 | 77% |
| 180 | 40281 ± 9427 | 28% |

Table 1 showed that the *in vitro* molecular weight changed over time (15, 30, 60 and 180 days) of the PLCL stents. The data indicated that the molecular weight started to decrease after the start of degradation study and retained 28% of the original molecular weight (143319 ± 4282 Da) at the end of the study.

.

**2.2 Radial strength changes**

**Table 2. Radial strength changes during real-time degradation (n=5)**

| **Degradation Time (Days)** | **Radial strength ± SD (mmHg)** | **Percentage (%)** |
| --- | --- | --- |
| 0 | 652 ± 51 | 100% |
| 15 | 1132 ± 90 | 174% |
| 30 | 1182 ± 141 | 181% |
| 60 | 1150 ± 91 | 176% |
| 180 | 169 ± 50 | 26% |

Table 2 showed the radial strength change of PLCL stents during the real-time degradation study. With the real-time degradation process going on, the radial strength of the stents increased gradually within the first month and then started to decrease in the second month.
